# Amalgam Work

**Published:** 1859-09

**Authors:** N. B. Slayton

**Affiliations:** Madison, Indiana


MR. EDITOR,

SIR :—You will do me a favor if you will grant me a small space in your journal to reply to an article written by Dr. Hunter on *Amalgam work.*

But in order that the profession may fully understand the position Dr. Hunter has taken, and his motives in writing that article, it is necessary I should first state of what I claim to be the inventor. The principal object of the amalgam is to fasten teeth to a gold plate, with or without soldering. I strike my plate and fit it to the mouth as usual, arrange my teeth on the plate as you would for backing, then I bend my rivets in a dovetail shape, or back my teeth with a narrow backing riveted on or soldered on as the dentist thinks best. I now make an amalgam of gold of any fineness, which I work in and around the rivets of the teeth or around the backings, filling up all lodgments for food, making a solid mass of gold as perfect as if cast, and nearly as solid. I at the same time work a rim out on my plate, building out or filling up where I choose. When this is completed to my satisfaction, I drive off all the mercury by heat, leaving nothing but the gold; this style of work has many advantages over ordinary gold work, it is stronger, neater, and more cleanly than work can possibly be done by the ordinary method.

It does not admit the fluids of the mouth any more than continuous gum work; it is harder than gold in the coin, but not so dense; it will stand a high finish. I have made at least thirty pieces of this work since last March, some of the teeth have been soldered on, and some only the pivots slightly bent; both have stood the test equally well. I have had only two teeth that has come off in that time, and that was owing to the shortness of the rivets. I have made application for a patent for my improvement, and expect to get one. I ask *no* dentist to use my improvement, but if they wish to do so, I charge them a reasonable sum for an office right; I have published nothing on the subject nor ask any one to do so, neither have I sent circulars to the profession. Two or three of the journals have kindly spoken of the work, but very cautiously, and in no case have they recommended it to any one; they could not consistently say less than they did, and *more* I did not ask. But in every case I have requested them to condemn it, if it did not stand the test for all that was claimed for it.

It is unnecessary for me to enter into any argument with Dr. Hunter about the merits of the work he has described in his article, for unfortunately for Dr. Hunter, in no important point does he describe my work. The process he has given, the manner of preparing and using gold is *entirely useless;* but, when prepared as I prepare it, and worked as I work it, it accomplishes all I claim. It looks very strange to me that a man *pretending* to occupy the position that Dr. Hunter does, should come forward and publish an article condemning a thing that he does not understand even the first principles. I can't *understand* the Dr's motives—the most favorable construction I can give to it is, that he is *crazy.* I am afraid, however, it is *nature,* and he *can't help* it. No man in the profession has been so badly abused and so little appreciated in their own estimation, as Dr. Hunter. Some one is constantly robbing him of his inventions; all improvements in dentistry for the last fifteen years, has originated with him, but the Dr. only meets the fate of all *great inventors.* Some one is constantly stealing "his thunder."

The Dr. has very kindly given me the credit of being the inventor of gutta percha, but at the time it was first introduced he claimed to be the inventor and had used gutta percha for two or three years with good success, and was satisfied it would stand all I claimed for it. From Dr. Hunter I got the first written article in its favor, and to him I give the credit for all the benefit I received from it.

Now if the Dr. is anxious to know how to work amalgam, in order that his next article may contain some truth, and not all *guess work*, I will have him properly instructed in the *modus operandi.* I thank the Dr. for his advertisement, it is the best that I have had. N. B. SLAYTON,
Madison, Indiana.

